# The Effects of Omega-3 Supplementation on the Omega-3 Index and Quality of Life and Pain Scores in Dogs

**DOI:** 10.3390/ani14213108

**Published:** 2024-10-29

**Authors:** Carolina Carlisle, Brandon T. Metzger, Nathan L. Tintle, Kristine Polley, Kristina H. Jackson, Sara Le Brun-Blashka, Jody Griffiths, William S. Harris

**Affiliations:** 1 Nutrition Innovation Center, Standard Process Inc., 150 N Research Campus Dr, Kannapolis, NC 28081, USAjgriffiths@standardprocess.com (J.G.); 2 The Fatty Acid Research Institute, Sioux Falls, SD 57106, USA; nlt@faresinst.com (N.L.T.); kristina@omegaquant.com (K.H.J.); wsh@faresinst.com (W.S.H.); 3 Department of Population Health Nursing Science, College of Nursing, University of Illinois—Chicago, Chicago, IL 60612, USA; 4 OmegaQuant Analytics, LLC, Sioux Falls, SD 57106, USA; 5 Department of Internal Medicine, Sanford School of Medicine, University of South Dakota, Sioux Falls, SD 57105, USA

**Keywords:** docosahexaenoic acid, eicosapentaenoic acid, Omega-3 Index, canine, dog, pain, quality of life

## Abstract

This study in dogs had two objectives: first, to determine whether a daily supplement of marine omega-3 (EPA and DHA) would (1) increase red blood cell EPA + DHA levels (i.e., the Omega-3 Index derived from dried blood spot analysis) and (2) impact health-related measures. Dogs were recruited from five private veterinary clinics in the United States and supplemented with about 70 mg of EPA + DHA per kg body weight (BW) for 16 weeks. Health-related outcomes included quality of life (QOL) and pain scores using validated questionnaires. Twenty-nine dogs (8.4 ± 3.6 years old) finished this study. After 16 weeks of supplementation with 68 ± 19 mg EPA + DHA/kg/day, the O3I increased from 1.4% ± 1.0% at baseline to 3.3% ± 1.1% (*p* = <0.0001). Concomitantly, overall pain scores declined from 7.4 ± 5.8 to 6.2 ± 5.4 (*p* = 0.012), primarily in small and medium-sized dogs. QOL scores improved slightly but only in small dogs (*p* = 0.03). Thus, supplementation with about 70 mg of EPA + DHA/kg/day improved the O3I and, in smaller dogs, pain and QOL scores. Future studies are needed to more clearly define doses of EPA + DHA that would improve the health of dogs.

## Simple Summary

There is a growing interest in the impact of omega-3 fatty acids [i.e., eicosapentaenoic acid (EPA) and docosahexaenoic acid (DHA)] on the health of dogs. The purpose of this study was to understand the effects of EPA + DHA supplementation in dogs on, first, the erythrocyte EPA + DHA level (i.e., the Omega-3 Index) and, second, health-related outcomes. We found that supplementation significantly improved the Omega-3 Index in all dogs, pain scores in small and medium-sized dogs, and quality of life scores in smaller dogs. Some of these effects may have been dose-related. Overall, our findings support the hypothesis that increased EPA + DHA intake in dogs fed standard commercial diets may provide health benefits, but further research is needed to define target Omega-3 Index levels associated with specific health conditions and optimal doses.

## 1. Introduction

Understanding the role of the marine omega-3 fatty acids (n-3 FAs), namely EPA and DHA, in canine health is an important research and clinical project. Bauer reviewed this field in 2016, making the case that, although several studies have reported therapeutic effects in a variety of conditions, more research was needed to define the optimal dose for any given disease [1]. A growing body of research has examined the effects of supplementation of omega-3s FA in dogs, with systematic reviews reporting some benefits in a variety of diseases/conditions [2,3] or on serum disease biomarkers [4]. Some studies report positive associations between n-3 FA supplementation and health-related outcomes [2,5] while others find no effect [6,7,8,9]. Some of this diversity likely derives from differences in study designs, including the doses of EPA + DHA administered, the durations of treatments, the background diets, the baseline health status of the dogs, and the choice of circulating biomarkers measured [2,10,11]. This heterogeneity makes it difficult to draw firm conclusions about the role of omega-3 FAs in canine health, and thus further research is needed.

There were two objectives of this study. First, we aimed to determine whether daily supplementation of about 70 mg of EPA + DHA per kg of body weight (BW) per day for 16 weeks would significantly increase the n-3 FA profile (EPA and DHA) in red blood cells (RBC) in dogs (i.e., the Omega-3 Index—O3I). The second aim of this study was to determine whether this dose would significantly impact quality of life (QOL) and pain scores.

## 2. Materials and Methods

### 2.1. Study Design, Ethics, and Subjects

This was an open-label study in healthy companion dogs. The study protocol was reviewed and carried out by the attending veterinarians and client-owned animals were treated as routine patients [12]. Participants were recruited through flyers describing the goals of the research in local veterinarian clinics and enrolled from five cities throughout the US (Severna Park, MD; Montgomery, IL: Hillsborough, NC; Lima, OH; and Oakland, CA). All dogs in this study were privately owned and under the care and supervision of a licensed veterinarian. Owners were informed about this study, its procedures, and the study product. Each dog’s owner signed an informed consent form before enrollment that outlined the study procedures. This was in accordance with recommendations from the FDA’s Center for Veterinary Medicine (CVM) regarding studies with client-owned animals. Owners also gave their permission to use the data for research purposes. In addition, this study was conducted in accordance with Good Clinical Practice Guidelines. For these reasons, the Animal Ethics Review Committee believes that this study did not specifically require review prior to being conducted.

Inclusion criteria for this study were generally healthy dogs of both sexes, at least 3 years of age, and not taking an n-3 FA/fish oil supplement in the past 6 months. We excluded any dog that (a) had any previously diagnosed illnesses such as cancer, kidney disease, or diabetes; (b) was receiving daily anti-inflammatory medications; (c) was consuming fresh/home-cooked fish weekly; (d) was consuming any fresh pet food with fish as primary protein; (e) was consuming fish-based kibble daily; (f) was pregnant or nursing; and (g) had known allergies to fish (like sardines/anchovies).

The dog’s date of birth, age, BW, breed, current diet (commercial product’s brand name and daily amount), and supplement/medication use were recorded at baseline. Additionally, the owners were asked to maintain their dog’s current diet and supplements/medications throughout this study.

### 2.2. Intervention

Owners were provided with the EPA + DHA supplement in soft gel format (Standard Process Inc., Palmyra, WI, USA). Each 1 g soft gel contained 250 mg of EPA and 200 mg of DHA sourced from anchovy and sardine oils. Owners were instructed to administer about 70 mg of EPA + DHA/kg/day based on the dog’s body weight (BW) [2,13] for 16 weeks. For example, for a dog weighing 20 kg, the target dose would 1400 mg of EPA + DHA. At 450 mg/capsule, this would compute to 3.1 capsules per day. The use of fixed-dose capsules therefore required flexibility on the actual mg/kg dose; for a 20 kg dog, it would be 67.5 mg/kg/day. In practice, one additional soft gel was prescribed for every additional 9 kg. The approximate dose was about 70 mg EPA + DHA per kg BW. This dose was similar to that provided in previous canine studies [4,14].

Each owner was instructed on the specific number of capsules to be fed daily. A paper calendar was provided to the dog’s owner to record compliance with the intervention as a reminder. They were instructed to return the calendar and the supplement bottles at the end of this study. The owners were also asked to report any adverse events that may have occurred. All supplements and assessments were provided at no cost to the owner. All physical examinations and blood collections were performed at the veterinary offices based on current guidelines and in the presence of the dog’s owner.

### 2.3. Analyses of Omega-3 FA Profile

The Omega-3 Index (O3I) was used to assess the dogs’ omega-3 FA status in a similar manner as hemoglobin A1c, which serves as a biomarker of glycemic status. The method has been validated for dried-blood-spot (DBS) analysis from dogs by comparing the FA profile of DBS and RBC samples from 33 animals as recently reported [15]. O3I collection kits were provided to the veterinarians for use at baseline and at the end of this study (16 weeks). The kits included a sample collection card, instructions, and a pre-paid envelope to mail the sample to the laboratory for analysis (OmegaQuant Analytics, LLC., Sioux Falls, SD 57106, USA).

### 2.4. Dried-Blood-Spot and Laboratory Analyses

Blood samples were collected by the veterinarian at baseline and 16 weeks by foreleg venipuncture. After blood collection, the veterinarians were instructed to place 1–2 drops of blood (~50 µL) from the blood tube onto the sample collection card, let it dry for 15 min, and then put the sample in the mail as per kit instructions. All samples were received in the laboratory and analyzed upon receipt. The analytical method has been published previously [16]. Briefly, a punch was taken from the DBS cards and heated at 100 °C with methanol, toluene and boron trifluoride to produce FA methyl esters. These were then extracted into hexane and analyzed by gas chromatography (GC-2010, Shimadzu Corp, Columbia, MD, USA) using a 100 m SP2560 capillary column, where FAs were identified by comparison with known standards.

### 2.5. Health-Related Outcomes

The impact of EPA and DHA on health-related outcomes was measured by assessing pain and overall QOL at baseline and after 16 weeks. These outcomes were selected based on literature reports of musculoskeletal conditions being the most common disorders in dogs, resulting mostly in lameness and pain [17,18] and impacting QOL [19]. Each dog’s QOL was assessed using the Canine Health-related Quality of Life Survey (QLS), which is an owner-based validated instrument [20]. The questionnaire contains four health-related domains (happiness, physical functioning, hygiene, and mental status) that are scored on a 5-level Likert scale (1 = disagree to 5 = agree), two open-ended questions on general health that are scored in a 5-level Likert scale (1 = worse to 5 = better), and one direct question of the dog’s QOL as perceived by the owner measured on a 10-point scale (0 = “poor” to 10 = “excellent”). Dog owners typically completed the QLS in 10 min or less. A computer-generated QOL score for each domain was determined by the sum of the item scores, with a higher score indicating a better overall QOL [20]. Pain was measured using the Helsinki Chronic Pain Index (HCPI) for dogs which is a validated owner-based instrument to assess pain, physical functioning, and behavior [21]. The HCPI total score is constructed as the sum of answers to 11 questions that are scored on a 5-level Likert scale (0 = highest possible to 4 = lowest possible). Total scores range from a minimum of 0 and to a maximum of 44. Lower index scores indicated less pain [21].

### 2.6. Statistical Analysis

The sample size of this study was calculated based on guidelines in the literature for studies with repeated measures [22], with an alpha of 0.05 and a power of 0.80 to detect an effect on the primary endpoint, the O3I. This calculation indicated a minimum sample size of 20 dogs. In addition, a 20% attrition rate was incorporated into this study’s final sample size.

Unless otherwise indicated, all data are reported as means and standard deviations. Initially, a Shapiro–Wilk test assessed the normality of the data. Normally distributed data were analyzed using a paired *t*-test; otherwise, Wilcoxon signed-rank tests were used.

Because the dosage of the daily supplement varied by BW, we stratified our analyses by weight. Dogs weighing < 24 lbs (<11 kg) were considered “small”, dogs weighing between 25 and 59 lbs (12–27 kg) were considered “medium”, and those weighing 60 lbs or more (>28 kg) were considered “large”. Repeated measures ANOVA was used (if the data were normally distributed) or if not, by Kruskal–Wallis (non-parametric) analyses. To explore the relationship among the study variables, bivariate correlation coefficients (Pearson’s or Kendall’s Tau) were calculated. Non-parametric Kendall’s Tau (τ) was selected because of its greater accuracy for small datasets as compared to Spearman’s rho correlations. All analyses were computed using SAS JMP^®^ v.15 (Cary, NC, USA) and a two-tailed *p*-value less than 0.05 was considered statistically significant.

## 3. Results

### 3.1. Characteristics

Thirty dogs (15 of each sex) were enrolled in this study, six from each of five participating veterinary clinics. Of these, one female dog did not complete the baseline assessment and was excluded from the final analyses. Twenty-nine dogs completed this study. The majority of dogs (~70%) were considered to be ‘one-breed’ by owners and were neutered or spayed (90%) (Table 1). Dog breeds varied in terms of sizes and included small dogs such as Chihuahuas, medium dogs such as beagles, and large dogs such as Labradors. All dogs were adults (≥3 years old) and the average age was 8.4 years. The average daily dose of EPA + DHA was 68 mg/kg with no evidence of variation by sex (*p* = 0.84) or age (*p* = 0.87). All dogs were fed commercial dog food.

### 3.2. Overall Outcomes

The effects of supplementation on body weight, the O3I, and pain and QOL scores are shown in Table 2. There was no change in BW over the 16-week study; however, there were statistically significant changes in the O3I (+136%) and pain scores (−16%), but not in QOL scores.

### 3.3. Outcomes by Size Category

Outcomes stratified by size category are reported in Table 3. Small dogs received 1 to 2 capsules of the study supplement (450 mg–900 mg of EPA + DHA), medium-size dogs received 2 to 3 capsules of the study supplement (900 mg–1350 mg of EPA + DHA), and large-size dogs received 4 to 5 capsules of the study supplement (1800 mg–2250 mg of EPA + DHA). Mean doses per group are shown in Table 3. Large dogs received a significantly lower dose than small and medium dogs (whose doses were similar); however, effects on the O3I did not differ significantly by group. No adverse events were reported by the owners, and no dogs dropped out during this study.

Pain scores were significantly reduced by 38% in small dogs (*p* = 0.015) and by 30% in medium dogs (*p* = 0.007). There was no reduction in pain scores in large dogs (*p* = 0.38). Although overall QOL scores did not improve (Table 2), they did in small dogs (*p* = 0.03) (Table 3).

Finally, bivariate nonparametric correlation analyses (Kendall’s Tau) were computed to assess the relationship between O3I and health-related outcomes. There was a significant (*p* < 0.0001) inverse relationship between the pain and QOL scores at the end of the intervention (*rτ* = −0.60). However, there were no significant correlations between (post) O3I and (post) QOL (*rτ* = 0.16) or (post) pain scores (*rτ* = −0.16) (*p* = 0.2 both).

## 4. Discussion

The purpose of this study was twofold; first, to determine whether a dose of about 70 mg of EPA + DHA/kg/day would significantly increase the O3I in healthy dogs, and second, whether it would impact two health-related metrics, pain and QOL.

### 4.1. Effects on the Omega-3 Index

The average dosage used in this study (68 mg of EPA + DHA/kg) was similar to that used in at least three previous studies [4,14,23]. This dosage falls within the range suggested by current National Research Council (NRC) guidelines [24] which recommend a minimum of 30 mg of EPA + DHA/kg for adult dogs to maintain their metabolic functions and up to 370 mg/kg to impact health-related outcomes. To our knowledge, there have only been a few previous studies examining the effects of omega-3 supplementation on the O3I in dogs. Burri et al. fed Alaskan huskies a diet enriched with krill oil (8% by weight; exact dose of EPA and DHA is unknown) and observed a 19% increase in the O3I after 5 weeks [25]. Another study in huskies reported a 60% increase in the O3I [11]. In our study, supplementation with EPA + DHA for 16 weeks more than doubled the O3I (+135%) which is greater than that seen in past studies [10,11,13]. For instance, Burri et al. [10] found that 1.7 g of EPA + DHA over 6 weeks increased the O3I by 20% in the fish oil group but by 62% in the krill oil group. Similarly, Lindqvist and colleagues [13] reported a 21% increase in the O3I after supplementing dogs with about 450 mg of EPA + DHA for four weeks [13]. Omega-3 FA levels take about 12-16 weeks to reach a steady state [26]; thus, short periods of supplementation will lead to smaller impacts on the O3I. Finally, higher doses of EPA + DHA produce dose-dependent increases in the O3I [27].

### 4.2. Effects of EPA + DHA on Health Outcomes

In this study, EPA + DHA reduced overall pain scores by 19%, largely due to changes in small and medium dogs. This finding was most likely due to the anti-inflammatory properties of EPA + DHA. Eicosanoid and docosanoid (lipid-based signaling molecules associated with innate immune responses) metabolites of EPA and DHA, respectively, tend to be anti-inflammatory as compared to those derived from arachidonic acid (AA) [28]. Both EPA and DHA play an important role in the suppression of inflammatory responses by competing with AA for receptor availability [29]. With increasing EPA + DHA, there is an inhibition of AA metabolism, resulting in a downregulation of the inflammatory sequence [30]. Although inflammation was not directly assessed in this study, pain and inflammation are closely related. Pain is one of the primary symptoms associated with inflammatory diseases in dogs, such as osteoarthritis, and reducing the inflammatory products of enzymes such as COX-2 is a common aim of managing the signs of pain associated with osteoarthritis in dogs [31]. Therefore, the significant decrease in pain observed here is likely the result of EPA + DHA damping down inflammatory processes [32]. The lack of effect on pain scores in the large dogs could be explained by a lower per kg dose of EPA + DHA.

The lack of significant effect on QOL scores could be explained by the fact that the dogs in this study were already generally healthy, with the majority of owners reporting high QOL scores at baseline (8.9/10) with small variation within groups (SD = 1.0). The statistically significant improvement in small dogs but not in the larger groups cannot be explained by the dose (which was the same in medium dogs) and may be a chance finding.

As noted earlier, the NRC recommends two different types of dosages of EPA + DHA supplementation, one for targeting health maintenance (30 mg/kg/day) and one for disease prevention (370 mg/kg/day). Interestingly, another study in dogs reported that approximately 104 mg/kg/day of EPA + DHA was not enough to positively impact lipid levels in hyperlipidemic dogs [33]. Another study reported that although 95 mg EPA + DHA/kg/day did not affect the expression of matrix metalloproteinases (enzymes responsible for the degradation of the extracellular matrix, including collagens and elastins) in synovial fluid in surgically treated knees, it did reduce their expression in the contralateral knee [34].

In a previous study in huskies, an increase in the O3I from 2.5% to 3.07% (recalculated from the originally reported O3I of 5.2% and 6.2% based on new equations to convert DBS EPA + DHA to the O3I in dogs [15]) was significantly correlated with lower inflammation in dogs [25], and, in a another study, an O3I of 4% was associated with significant improvements in dogs’ joint health [14]. Thus, an O3I of 3% or higher appears to be linked with improved health outcomes [35], which is supported by the results of this study.

### 4.3. Strengths and Limitations

The strengths of this study included the use of BW-adjusted dosing (in a manner that could be used at home by owners using encapsulated omega-3 products) and the use of validated pain and QOL scoring systems. We also used newly described conversion equations to derive the RBC EPA + DHA value (i.e., the O3I) from the DBS analysis. In addition, conducting this study in a real-world setting should help with external validity. Limitations included the lack of a placebo group, the relatively small sample size, and uncontrolled home diets. These factors should be addressed in further research.

## 5. Conclusions

Our findings support the growing body of evidence that EPA + DHA supplementation can favorably affect the health of dogs. Although it was not the intention of this study to define an optimal O3I for dogs, our data suggest that future studies on omega-3 fatty acids and canine health might target an O3I of >3% as a tentative threshold for improved health metrics. It is possible that dogs would benefit from higher dosages of EPA + DHA, which would naturally result in higher O3I values, but further research would be needed to test this.

## Figures and Tables

**Table 1:** Characteristics at baseline (*n* = 29).

	*n* or Mean (SD)	%
Age (years)	8.38 ± 3.58	
Sex		
Male	15	51%
Female	14	49%
Breed		
One-Breed	20	69%
Mix-breed	9	31%
Size		
Small (≤11 kg)	11	41%
Medium (12–27 kg)	8	24%
Large (28–45 kg)	10	34%
Spay/Neuter Status		
Yes	26	90%
No	3	10%

**Table 2:** Study measures at baseline (Pre) and at the end of this study (Post).

	Pre		Post		
	Mean	SD	Mean	SD	*p*-Value
Body weight (kg)	18.2	12.3	18.5	12.3	0.234
Omega-3 Index (%)	1.4	1.0	3.3	1.1	<0.0001
Pain Score	7.4	5.8	6.2	5.4	0.012
Quality of Life	8.9	1.0	9.1	0.8	0.081

**Table 3:** Means (SD) of EPA + DHA dose and of Omega-3 Index, quality of life, and pain scores at baseline (pre) and after 16 weeks (post) by size category.

	Small Dogs *n* = 11		Medium Dogs *n* = 8		Large Dogs *n* = 10	
Dose (mg/kg/d)	76 (16) *		75 (22) *		53 (11)	
	Pre	Post	Pre	Post	Pre	Post
Body weight (kg)	7.2 (2.0)	7.2 (2)	15.0 (6.0)	15.5 (6.5)	33.0 (4.3)	33.6 (4.8)
Omega-3 Index%	1.5 (1.0)	3.1 (1.0) ^†^	1.7 (1.2)	4.1 (1.8) ^†^	1.1 (0.5)	3.0 (1.2) ^†^
Pain Score	6.0 (4.3)	3.7 (3.7) ^†^	9.4 (7.8)	6.5 (6.2) ^†^	7.5 (5.6)	8.7 (5.7)
Quality of Life	9.0 (1.0)	9.3 (0.7) ^†^	8.7 (1.4)	9.0 (0.8)	8.9 (1.0)	8.9 (0.8)

* *p* < 0.03 vs. large dogs; ^†^ *p* ≤ 0.05 post vs. pre.

## Data Availability

Raw data are potentially available upon request to the authors.
